# Complete Genome Sequence of a Chinese Strain of “*Candidatus* Liberibacter asiaticus”

**DOI:** 10.1128/genomeA.00184-13

**Published:** 2013-05-02

**Authors:** Hong Lin, Cliff S. Han, Binghao Liu, Binghai Lou, Xianjin Bai, Chongling Deng, Edwin L. Civerolo, Goutam Gupta

**Affiliations:** USDA-ARS San Joaquin Valley Agricultural Research Science Center, Parlier, California, USAa;; Los Alamos National Laboratory, Los Alamos, New Mexico, USAb;; Guangxi Citrus Research Institute, Guilin, Guangxi, Chinac;; Guangxi Academy Sciences of Agriculture, Nanning, Guangxi, Chinad

## Abstract

We report here the complete genome sequence of “*Candidatus* Liberibacter asiaticus” (strain Guangxi-1). The 1,268,237-bp genome with a 36.5% G+C content comprises 1,141 open reading frames, 44 tRNAs, and 3 complete rRNAs in a circular chromosome.

## GENOME ANNOUNCEMENT

Huanglongbing (HLB, or citrus greening) is the most devastating disease of citrus in the world (1). The disease is associated with three phloem-restricted, insect-transmitted, Gram-negative, and fastidious species of alphaproteobacteria in the genus “*Candidatus* Liberibacter.” The name of each species associated with HLB was based on its presumptive origin. “*Candidatus* Liberibacter asiaticus” is believed to have originated in Asia, “*Ca*. Liberibacter americanus” in the Americas, and “*Ca*. Liberibacter africanus” in South Africa (2, 3). Among these three species, “*Ca*. Liberibacter asiaticus” is the most widely distributed and responsible for the most significant economic losses to citrus production worldwide. The widely held assumption is that HLB originated in Asia (India or China). It was not until recently that HLB was introduced to the Western Hemisphere. HLB was reported in Brazil in 2004 and in the United States (Florida) in 2005 (4–6). Since then the disease has been observed in most citrus-growing counties in Florida and has been reported in other citrus-producing states, including Texas and California. Recent introduction of “*Ca*. Liberibacter asiaticus” into the Americas sparked research interest in understanding the epidemiology of HLB in relation to the genomic evolution of HLB-associated “*Ca*. Liberibacter” species, the origin of the disease, and the adaptive potential and pathogenic diversity of Asian and American strains of “*Ca.* Liberibacter asiaticus.”

Here we report the complete genome sequence of a Chinese strain of “*Ca*. Liberibacter asiaticus,” strain Guangxi-1 (GX-1). Genomic DNA was isolated from an Asian citrus psyllid (*Diaphorina citri*) collected from Guangxi, China. The complete genome sequence of the strain was obtained by using Illumina HiSeq 2000 with a 300-bp paired-end library, which achieved 50 to 80× coverage. *De novo* assembly was performed using the Velvet assembler version 1.1. Thirty-two contigs were identified via BLASTn analysis to belong to “*Ca*. Liberibacter asiaticus” sequences. The contigs’ alignment was aided with an available “*Ca*. Liberibacter asiaticus” genome of the psy66 strain (7). The contig gaps were then closed by PCR-based primer walking (8), and gap-closing PCR amplicons were resequenced using the Sanger sequencing method. The final annotation was reconfirmed by the NCBI Prokaryotic Genomes Automatic Annotation Pipeline (PGAAP) (http://www.ncbi.nlm.nih.gov/genomes/static/Pipeline.html). The genome of strain GX-1 comprises 1,268,237 nucleotides, a G+C content of 36.5%, 1,141 predicted coding sequences, 44 tRNAs, 3 complete copies of rRNA genes (16S, 23S, and 5S), and 368 hypothetical genes. Comparative analyses showed that the overall distribution of predicted genes based on functional categories for the GX-1 genome was nearly identical to that for the previously sequenced “*Ca*. Liberibacter asiaticus” psy66 strain. However, in contrast to the reported “*Ca*. Liberibacter asiaticus” strain psy66 genome, the GX-1 genome was found to have two tandem prophage segments. The average G+C content in this prophage region is ~40%, a value that is significantly different from that for the core genome. The GX-1 prophage sequence has 96 to 99% similarity to the “*Ca*. Liberibacter asiaticus” strain UF506 (9). The elucidation of this additional “*Ca*. Liberibacter asiaticus” genome sequence permits comparative genome analysis and advances understanding of genomic evolution in “*Ca*. Liberibacter.”

### Nucleotide sequence accession number. 

The completed genome sequence of “*Ca*. Liberibacter asiaticus” strain GX-1 has been deposited in the GenBank database under the accession number CP004005.
